# Pregnancy after Bariatric Surgery: Nutrition Recommendations and Glucose Homeostasis: A Point of View on Unresolved Questions

**DOI:** 10.3390/nu15051244

**Published:** 2023-03-01

**Authors:** Silvia Burlina, Maria Grazia Dalfrà, Annunziata Lapolla

**Affiliations:** Department of Medicine—DIMED, University of Padova, Via Giustiniani n 2, 35122 Padova, Italy

**Keywords:** bariatric surgery, pregnancy, nutrition

## Abstract

Obesity is increasing in all age groups and, consequently, its incidence has also risen in women of childbearing age. In Europe, the prevalence of maternal obesity varies from 7 to 25%. Maternal obesity is associated with short- and long-term adverse outcomes for both mother and child, and it is necessary to reduce weight before gestation to improve maternal and fetal outcomes. Bariatric surgery is an important treatment option for people with severe obesity. The number of surgeries performed is increasing worldwide, even in women of reproductive age, because improving fertility is a motivating factor. Nutritional intake after bariatric surgery is dependent on type of surgery, presence of symptoms, such as pain and nausea, and complications. There is also a risk of malnutrition after bariatric surgery. In particular, during pregnancy following bariatric surgery, there is a risk of protein and calorie malnutrition and micronutrient deficiencies due to increased maternal and fetal demand and possibly due to reduction of food intake (nausea, vomiting). As such, it is necessary to monitor and manage nutrition in pregnancy following bariatric surgery with a multidisciplinary team to avoid any deficiencies in each trimester and to ensure the well-being of the mother and fetus.

## 1. Introduction

Obesity is increasing in all age groups and, consequently, its incidence has also risen in women of childbearing age. In Europe, the prevalence of maternal obesity varies from 7 to 25% [1].

Obesity is associated with short- and long-term complications for both the mother and the child [2,3]. As for short-term complications for the mother, obese women are more likely to have early pregnancy loss, gestational diabetes mellitus (GDM), pregnancy-induced hypertension, cesarean section, thrombosis, post-partum hemorrhages, and infections. On the other hand, long-term health consequences are associated with weight retention after delivery, which increases future cardiometabolic risk [2,3,4].

As regards short-term complications for the child, there is an increased risk of congenital malformations, being large for gestational age (LGA), macrosomia, shoulder dystocia, premature birth, and stillbirth [5,6]. In addition, babies of obese women have a higher risk of developing obesity in childhood, and this increases the chance of type 2 diabetes and cardiovascular disease into adulthood [5,6,7].

As such, it is highly recommended that obese women lose weight before conception to improve maternal and fetal outcomes [8,9,10]. Lifestyle and pharmacological interventions are the main cornerstones for weight loss; however, in the case of patients with class III obesity (BMI ≥ 40 kg/m^2^) and patients with class II obesity (35–39 kg/m^2^) with associated comorbidities, bariatric surgery (BS) has proven to be effective [10].

Obese women who undergo BS need to be informed that the probability to become pregnant after BS is increased. In particular, pregnancies are not recommended shortly after BS and need to be planned after the phase of maximum weight loss due to the possible occurrence of micronutrient deficiencies. The consequences of micronutrient deficiencies on offspring well-being are not well-studied. International guidelines recommend conception at least 12 to 18 months after BS or until stabilization of weight after BS occurs [11,12].

After BS, it is necessary to manage pregnant women with a multidisciplinary team, especially to prevent micronutrient deficiencies for offspring.

In this paper, we aim to summarize the available evidence regarding the micro- and macronutrient needs of pregnant women after BS. In addition, we aim to consider unresolved questions in the management of women after BS, including maternal weight gain recommendations during pregnancy and glucose homeostasis.

## 2. Micronutrient Needs and Their Monitoring (Table 1)

All guidelines recommend multivitamin and mineral supplementation prior to conception and throughout pregnancy. In particular, biochemical assessment to determine the specific micronutrient needs for women after BS, both before and during pregnancy, is strongly advised [13].

**Table 1:** Recommended intake of micronutrients and vitamins in physiological pregnancy and in pregnancy after bariatric surgery.

	DRI/RDA/AI	BS	AGB	Note
Folic acid	0.6 mg	0.4 mg–1 mg		4–5 mg in obese and diabetic women
Calcium	1000 mg	1200–1500 mg		
Copper	1 mg	2 mg	>1 mg	
Iron	27 mg	45–60 mg	>18 mg	
Selenium	60 µg	50–100 µg		
Zinc	11 mg	8–22 mg		
Vitamin A	10,000 IU	5000 IU–10,000 IU		Beta-carotene form in pregnancy
Vitamin B1	1.4 mg	<12 mg		
Vitamin D	600 IU	>1000 IU (40 mcg)		
Vitamin E	15 mg	15 mg		
Vitamin K	90 µg	90–120 µg		

BS: bariatric surgery; AGB: adjustable laparoscopic binding; DRI: dietary reference intakes; RDA: recommended dietary allowances; AI: adequate intake.

This supplement should contain the following at a minimum: copper (2 mg), zinc (15 mg), selenium (50 μg), folic acid (0.4–1 mg), iron (45–60 mg or >18 mg after adjustable gastric band), thiamine (>12 mg), vitamin E (15 mg), and beta-carotene (vitamin A, 5000 IU) [13].

The retinol form of vitamin A should be avoided during pregnancy due to a teratogenicity risk [14,15]

As for folic acid supplementation, current guidelines suggest that folic acid at a dose of 4 or 5 mg daily should be given to patients who remain obese or diabetic after BS during the periconception period and throughout the first trimester [16].

As for vitamin B12, it is suggested to provide in the preconception period a dose of 1 mg every 3 months via intramuscular depot injection and monthly during pregnancy [17,18]. Alternatively, oral supplementation (1 mg/day) can be used to increase the compliance of the patient.

All guidelines for women in pregnancy after BS recommend an iron supplement at a minimum dose of 45 mg elemental iron daily (>18 mg for adjustable gastric band); this should be increased as needed to maintain ferritin within normal range. Clinical recommendations also suggest iron infusion if oral iron supplementation is not sufficient [19].

Vitamin D should be supplemented to maintain a concentration of 50 nmol/L or greater with serum parathyroid hormone within normal limits, and if necessary, calcium should be added to maintain parathyroid hormone within normal limits [20].

It is recommended to check the following indices at least once per trimester and to use pregnancy-specific ranges for: serum folate; serum vitamin B12; serum ferritin (iron studies including transferrin saturation and full blood count); serum vitamin D with calcium, phosphate, magnesium, and parathyroid hormone; serum vitamin A; prothrombin time, INR, and serum vitamin K1 concentration; serum protein and albumin; and renal function and liver function tests [18].

## 3. Macronutrient Needs

As regards macronutrients, there are no clear guidelines. There are no specific recommendations as regards energy intake, carbohydrates (CHO), fat, or fluid; it is recommended to follow the Institute of Medicine (IOM) guidelines for pregnancy, even if they are not specific for pregnancy after BS [21]. As for macronutrient composition, it should be evaluated and monitored by a dietician throughout pregnancy to meet the metabolic demands of the pregnancy and to achieve fetal growth targets [17]. Energy requirements should be individualized on the basis of pre-pregnancy BMI, gestational weight gain (GWG), and physical activity level, with limitations on energy-dense foods if excessive GWG is identified.

Only for protein intake is there a particular recommendation: a minimal target for protein intake after BS of 60 g/day and up to 1.5 g/kg ideal body weight per day [11], but higher amounts of protein intake (up to 2.1 g/kg ideal body weight per day) may be required in individual cases [19]. These recommended supplementation guidelines after BS are based on the results of a prospective one-year observational study showing an inverse relationship between protein intake and lean tissue loss [22]

It is important to underline that in normal pregnancy, daily protein intake of 0.9 g protein/kg body weight in the second trimester and 1.0 g/kg in the third trimester is recommended [21].

In Table 2, recommendations from guidelines for general patients after BS, general pregnant women, and pregnant women after BS are summarized.

## 4. Glucose Homeostasis in Pregnancy after Bariatric Surgery

Previous bariatric surgery has benefits in pregnancy as well as harms for mother and child. Regarding benefits, pregnancies after BS are associated with lower risk of GDM development, of LGA or macrosomia, and of hypertensive disorders in pregnancy. As for possible risks, a higher risk of small for gestational age (SGA) infants is reported in the literature [23,24,25].

In a normal pregnancy, there is increased insulin sensitivity in the first trimester, while the second and third trimester are characterized by progression of insulin resistance to guarantee a higher glucose load and a normal growth. After BS, there is an improvement in insulin resistance that, on one hand, can explain the decreased risk of GDM development, but on the other hand, could explain the increased risk of SGA, because insulin resistance could be insufficient in the third trimester to provide enough glucose flux to the fetus.

It is important to underline that glucose homeostasis after BS depends on the type of surgical technique used. BS procedures can be classified as restrictive techniques (sleeve gastrectomy (SG)), malabsorptive techniques (intestinal bypass), or mixed (restrictive and malabsorptive: Roux-en-Y gastric bypass (RYGB)).

In particular, glucose homeostasis takes longer to improve in restrictive procedures (such as SG) [26]; on the other hand, a full type 2 remission is observed within days or weeks in the case of mixed procedures (such as RYGB) [27,28].

## 5. Diagnosis of Diabetes in Pregnancy after BS

Women undergoing BS often remain overweight or obese; as such, they are at high risk of developing type 2 diabetes and GDM. As such, it is necessary to diagnose preexisting diabetes or GDM to avoid maternal and fetal complications related to hyperglycemia in pregnancy. There are no specific guidelines or cut-off values for the diagnosis of preexisting diabetes or GDM in pregnant women after BS, so it is recommended to follow international guidelines [29]. The oral glucose tolerance test (OGTT) with 75 g glucose is the gold standard for the diagnosis of GDM [29], but in women after BS, there are concerns with regards to its tolerability and accuracy. A Consensus Recommendation has proposed the use of seven-point capillary blood glucose monitoring or continuous glucose monitoring (CGM) for one week between 24 and 28 weeks of pregnancy [18]. In women in remission of type 2 diabetes after BS, it is recommended to screen for diabetes with fasting plasma glucose or glycated hemoglobin at booking and in the second trimester [18]. There are no specific glycemic targets for pregnant women after BS complicated by GDM or type 2 diabetes, so it is recommended to use international or local targets [18], generally 95 mg/dL for fasting glucose and 140 mg/dL for glucose level one hour after a meal.

## 6. Postprandial Hyperinsulinemic Hypoglycemia

It is known that the gastrointestinal tract produces and secrets many polypeptides that play an important role in glucose homeostasis [30]. The more important hormones released by the gastrointestinal tract are incretins that stimulate postprandial insulin secretion in response to food intake. The two main incretins are the gastric inhibitory polypeptide (GIP) and the glucagon-like peptide-1 (GLP-1) [31]. Data available in the literature suggest that postprandial GLP-1 significantly increases, mainly in malabsorptive BS, but why this happens remains incompletely elucidated [32]. On the other hand, postprandial GIP levels are reduced after RYGB but are not changed after restrictive surgical procedures [33].

The increased secretion of GLP-1 is one candidate mediator of an important complication of BS, particularly RYGB: postprandial hyperinsulinemic hypoglycemia (PHH). PHH occurs in late dumping syndrome, typically within 1 to 3 h of ingestion of CHO-rich meals [34]. Rapid transition through the gastrointestinal tract stimulates L-cells and their secretion of GLP-1, followed by the increased secretion of insulin by the pancreatic beta-cells [35]. Recently, Larraufie et al. explored the importance of GLP-1 in post-BS secretion. They analyzed transcriptomics and peptidomics of enteroendocrine cells and demonstrated that elevated GLP-1 levels correlated with increased nutrient delivery to the gastrointestinal tract. They concluded that increased GLP-1 secretion after BS is the result of increased nutrient transit to the distal gut. They also demonstrated that GLP-1 is the major driver of insulin secretion [36]. In particular, GLP-1 level evaluation could be useful to prevent complications after BS, especially during pregnancy.

PHH can have few symptoms, which causes delayed or missed diagnosis. It is important to underline that at the moment there is no consensus on diagnostic criteria of PHH. Recently, confirmation of Whipple’s triad with symptoms (anxiety, sweating, altered sensorium, hunger, and syncope) at a glucose level below 54 mg/dL and remission of symptoms after restoration of glucose level has been proposed [37]. During pregnancy, hypoglycemia can be harmful for the mother and even for the fetus in terms of fetal growth restriction. Recent data suggest a correlation between hypoglycemia events and reduced birthweight in pregnancy after BS [38,39]. Recognition of hypoglycemia episodes in pregnant women after BS is necessary to prevent fetal complications, such as fetal growth restriction. Available provocation tests are the OGTT and the mixed meal tolerance test; however, at the moment, there is not an accepted standard meal test. Nutritional management of PPH is necessary and includes recommended ingestion of low-glycemic-index CHO, small CHO portions, frequent intake of food with at least six small meals a day, and ingestion of CHO mixed with protein [37] (Table 2).

Another common effect of BS is early dumping syndrome that occurs within 1 h of ingestion of food, especially rapidly absorbed CHO, and is characterized by the presence of symptoms such as dizziness, flushing, palpitations, and gastrointestinal symptoms (abdominal pain, diarrhea, and nausea). If suspicious of early dumping, rapidly absorbed CHO should be avoided, ingestion of liquid should be avoided 30 min before and after eating, and patients should be advised to eat slowly and to pay attention to portion size and meal frequency during the day [18] (Table 2).

## 7. Glucose Homeostasis Evaluation after BS

Data on glucose homeostasis in pregnancy after BS are few and heterogeneous. The majority of studies have utilized OGTT with 75 g or 100 g to evaluate glucose homeostasis. A high prevalence of hypoglycemia during OGTT is reported from 5.26% to 90% of all women evaluated [40]. In particular, it seems that pregnant women after RYGB have a glycemic raise at 60 min followed by hypoglycemia at 120 min in 54.8% of cases. If OGTT is extended to 180 min, 90% of women develop hypoglycemia.

When the relation between alteration of OGTT values and risk of SGA was evaluated, a positive association was found between fetal growth and maternal glucose nadir during OGTT [38]. In addition, an association between maternal hypoglycemic events during OGTT and SGA infants was found.

OGTT can be helpful to analyze glucose homeostasis after BS, but CGM could provide a more detailed analysis of glucose homeostasis, especially after BS. In fact, with CGM it is possible to find all glycemic excursions, all hypoglycemic events, and time spent in range, above range, or below range, and these data could help clinicians to better individualize macronutrient needs.

A recent meta-analysis has evaluated the rate and the timing of post-bariatric hypoglycemia (PBH) with CGM in non-pregnant patients [41]. The weighted mean prevalence of PBH was 54.3%, with a comparable rate of PBH in patients treated with RYGB and SG. The weighted mean prevalence of nocturnal PBH was 16.4%, with a comparable rate of PBH in patients treated with RYGB and SG. It was also found that the rate of PBH increased with increasing time from surgery.

Bonis et al. studied the CGM profiles of 35 pregnant women after RYGB. They found a CGM profile similar to that described in non-pregnant RYGB patients. In particular, CGM profile was characterized by high mean maximum glucose level (200 mg/dL) and low mean minimum glucose level (<50 mg/dL). The time to reach post-prandial glycemic peak was short (<60 min) [42].

Interestingly, Gohier et al. studied the CGM profiles of 122 pregnant women who had undergone RYGB surgery. They found that 73% of women had CGM profile abnormalities; in particular, 55% of the women spent a higher time over 140 mg/dL, and 68% of the women spent a higher time below 60 mg/dL. In addition, they demonstrated that CGM profile abnormalities are associated with fetal complications. In particular, being LGA was associated with a higher time spent over 140 mg/dL and with an excessive maternal weight gain during pregnancy; prematurity and being small for gestational age were associated with a higher time spent below 60 mg/dL [43].

It is important to underline that it is necessary to have more data to define better optimal glycemic targets in pregnant women after BS. Further studies on CGM in pregnant women after BS are necessary to find the target range of time to prevent maternal and fetal complications.

CGM could be a valid option in pregnancy after BS to better analyze glucose profile and, in particular, to find all hypoglycemic events, even without specific symptoms. These important data could help all clinicians and, in particular, dieticians to modify macronutrient intake to avoid hypoglycemic events that could be harmful to the fetus.

## 8. Conclusions

In conclusion, there are specific guidelines that clearly define micronutrient needs and their monitoring in pregnancies after BS. On the other hand, there are many unresolved questions in the management of nutrition in pregnancy after BS. In particular, it is necessary to better define the target of maternal weight gain during pregnancy. At the moment, IOM guidelines are used, but they are not specific to these patients because they do not consider weight loss after BS and the gap time between surgery and pregnancy. In addition, it is necessary to better define macronutrient needs in these women, taking into account the type of surgery (restrictive techniques, malabsorptive techniques, or mixed), prepregnancy BMI, recommended GWG, physical activity level, and the presence of PPH or early dumping syndrome. In this complex scenario, it seems that evaluation of hormones secreted by the gastrointestinal tract (particularly GLP-1 secretion) and analysis of glucose profiles of these women (particularly with CGM) could provide important information to answer these unresolved questions and to better individualize specific needs in order to prevent maternal and fetal complications associated with pregnancy after BS.

## Figures and Tables

**Table 2:** Macronutrient recommendations for general patients after BS, general pregnant women, and pregnant women after BS. CHO: carbohydrates; GI: glycemic index; BMI: body mass index.

	General Patients after BS [19]	General Pregnant Women [21]	Pregnant Women after BS [18]
Energy intake	No specific recommendation	+340 Kcal/day in the second trimester +452 Kcal/day in the third trimester	Individualized on the basis of pre-pregnancy BMI, gestational weight gain, and physical activity level
CHO	No specific recommendation	45–65% of total energy intake	If hyper- or hypoglycemia, modify CHO quantity or quality If dumping syndrome, avoid simple CHO, use protein and low GI CHO, and divide food into six small meals
Protein	60 g/day and up to 1.5 g/kg ideal body weight per day	10–35% of total energy intake	No specific recommendation; refer to recommendations for general patients after BS
Fat	No specific recommendation	20–35% of total energy intake	No specific recommendation
Fluid	No specific recommendation	3 L/day	No specific recommendation

## Data Availability

Not applicable.
